# Clonal expansion of *Mycobacterium tuberculosis* isolates and coexisting drug resistance in patients newly diagnosed with pulmonary tuberculosis in Hanoi, Vietnam

**DOI:** 10.1186/1756-0500-6-444

**Published:** 2013-11-05

**Authors:** Nguyen Van Hung, Hiroki Ando, Tran Thi-Bich Thuy, Tomoko Kuwahara, Nguyen Thi-Le Hang, Shinsaku Sakurada, Pham Huu Thuong, Luu Thi Lien, Naoto Keicho

**Affiliations:** 1 Department of Microbiology, National Lung Hospital, 463 Hoang Hoa Tham, Hanoi, Vietnam; 2 National Center for Global Health and Medicine, Tokyo, Japan; 3 NCGM-Bach Mai Hospital Medical Collaboration Center, Hanoi, Vietnam; 4 Hanoi Lung Hospital, Hanoi, Vietnam; 5 Hanoi Department of Health, Hanoi, Vietnam; 6 Research Institute of Tuberculosis JATA, Tokyo, Japan

**Keywords:** Primary drug resistance, Isoniazid, Gene mutation, Restriction fragment length polymorphism, Vietnam

## Abstract

**Background:**

Newly diagnosed patients without anti-tuberculosis (TB) treatment histories have not often undergone drug susceptibility testing (DST), but have received the standard treatment regimen without information about their DST profiles in many countries with inadequate resources.

**Methods:**

We collected 346 clinical isolates from previously untreated patients with smear-positive active TB in Hanoi, the capital of Vietnam. Of these, 339 were tested for susceptibility to four first-line anti-TB drugs, including isoniazid (INH), rifampicin (RMP), streptomycin (SM), and ethambutol (EMB), using the proportion method. A pyrazinamidase (PZase) test was used to assess pyrazinamide (PZA) resistance. Results of the culture-based drug susceptibility tests were confirmed by those from reverse hybridization-based line probe assays (LiPAs) that detected mutations associated with RMP, INH, PZA, and fluoroquinolone (FQ) resistance. To investigate a diversity of these strains, IS*6110-*probed restriction fragment length polymorphisms (RFLPs) were analyzed. Nucleotide sequences for *furA-katG* and *fabG1-inh*A operons, transcription units responsible for INH resistance, were also determined.

**Results:**

Of the isolates tested, 127 (37.5%) were resistant to at least one of the four drugs, which included 93 (27.4%) isolates that were resistant to INH. RFLP analysis identified four clusters defined by similarity of the band patterns, which accounted for 46.1% of the tested isolates. Among the clustered isolates, 37.7% were resistant to INH, most of which (85.4%) carried a g944c mutation, which causes an S315T amino acid substitution, in the *katG* gene.

**Conclusions:**

Our results suggest that drug-resistant strains, particularly those with INH resistance characterized by a single mutation, S315T, are spreading in Hanoi, Vietnam. When RMP resistance is combined with this setting, patients are not easily cured by conventional short-term treatment. We will need to carefully monitor these trends and search for the origins and transmission routes of these strains.

## Background

The drug susceptibility profiles of clinically isolated *Mycobacterium tuberculosis* (MTB) strains, particularly those from previously untreated patients, have not been included in clinical practice in many countries with inadequate resources. A single standard anti-tuberculosis (TB) treatment without information regarding drug susceptibility is prone to failure or relapse, as initial drug resistance increases the chance of acquiring additional drug resistance [1].

Molecular fingerprinting of MTB strains has been used extensively and is crucial for elucidating the transmission routes of drug-resistant TB [2,3]. A rapidly developing large city is often accompanied by overcrowding and a floating population, and it is often not easy to identify the epidemiological link between TB cases. Nevertheless, the molecular epidemiological techniques are useful for providing insights into the spread patterns of MTB on site and can thus aid in enhancing TB control activities in the entire city.

Vietnam is a Southeast Asian country stretching over 1,800 km from north to south. It is one of 22 high-burden countries worldwide, and its TB prevalence remains high (323 per 100,000 in 2011) [4]. Vietnam reported an incidence of 2.7% multi-drug resistant-TB among new cases in a 2006 survey (95% confidence interval: 2.0–3.6) [5].

The northern and southern regions of Vietnam have also been under different health policies for more than 20 years. It remains unclear whether entire profiles of MTB isolates obtained in one area are equally useful throughout the country. An earlier report [6] suggested differences in genotypes and drug susceptibility patterns between isolates obtained in distant regions of Vietnam.

Although the status of primary drug resistance has been reported in some areas of Vietnam [7-9], molecular biological approaches to this issue have not yet been completely exploited. Thus, we analyzed the profiles of drug susceptibility testing (DST), drug resistance genes, and fingerprint patterns of MTB isolates obtained from 339 previously untreated patients with smear-positive active TB in Hanoi, the capital of Vietnam.

## Methods

### Ethics statement

A written informed consent was obtained from each participant. The study was approved by ethical committees of the Ministry of Health, Vietnam and National Center for Global Health and Medicine, Japan.

### Clinical isolates from acid-fast bacilli (AFB)-positive sputum

Clinical isolates were consecutively collected from previously untreated patients with AFB-positive active TB in Hanoi city between August 2007 and August 2008. At least two sputum specimens were collected from each patient; one was for a smear test and the other was used for culture in the Department of Microbiology of the Hanoi Lung Hospital. Specimens were decontaminated and homogenized with 0.5% NALC–2% NaOH and subsequently inoculated on Löwenstein–Jensen media. MTB isolates were transferred to the Molecular Biology Laboratory of the National Lung Hospital and subjected to MTB identification using niacin and nitrate tests, DST, and other molecular epidemiological tests.

### Drug susceptibility testing (DST)

DST was performed using the proportion method based on World Health Organization (WHO) guidelines [10]. The test medium contained rifampicin (RMP; 40 μg/mL), isoniazid (INH; 0.2 μg/mL and 1.0 μg/mL), ethambutol (EMB; 2.0 μg/mL), and streptomycin (SM; 4.0 μg/mL). Drug resistance was defined as ≥1% colony growth compared with a drug-free control of Löwenstein–Jensen medium.

### Pyrazinamidase (PZase) assay

PZase activity was determined by Wayne’s method with minor modifications [11,12]. As a positive control, we used the MTB H37Rv strain that is susceptible to pyrazinamide (PZA) and is positive for PZase. As a negative control, we used the *M. bovis* BCG strain that is resistant to PZA and is negative for PZase.

### Isolation of genomic DNA

Genomic DNA from MTB was extracted using the original method described [13], with slight modifications [14]. Approximately 400 μl of a bacterial suspension in TE buffer was heated at 80°C for 20 min to kill bacteria. First, 50 μl of lysozyme (10 mg/ml) was added followed by incubation at 37°C for 1 h. Subsequently, 75 μl of SDS/proteinase K was gently mixed followed by incubation at 65°C for 10 min. In addition, 100 μl of 5-M NaCl and 100 μl of CTAB/NaCl solution were thoroughly mixed and incubated for 10 min at 65°C. An equal volume (approximately 750 μl) of chloroform/isoamylalcohol was added, and the mixture was centrifuged for 5 min at 12,000× g. The aqueous supernatant was carefully transferred to another tube. Total DNA was precipitated in isopropanol and was dissolved in 0.1× TE buffer.

### Line probe assays (LiPAs)

Reverse hybridization-based LiPAs were used to confirm the results of DST and to detect mutations associated with resistance to RMP [15], INH [16], PZA [12], and fluoroquinolone (FQ) [17]. To detect mutations associated with RMP resistance, 5 oligonucleotide probes were used to hybridize to wild-type sequences and 4 probes to mutation sequences of the *rpoB* gene. For INH resistance, 41 oligonucleotide probes were designed to cover mutations in the regions of *katG* (35 probes), *furA* (2 probes), *fabG1-inhA promoter* (2 probes), and *fabG1* (2 probes). The details and performances of these tests have been reported in the references described above [12,15-17].

### Restriction fragment length polymorphism (RFLP)

Experimental procedures for bacterial growth, DNA extraction, DNA digestion with *Pvu*II (Takara Bio Inc. Otsu, Japan), electrophoresis on a 1% agarose gel, and Southern blotting and membrane hybridization with a peroxidase-labeled 245-bp IS*6110* probe were performed using standardized methods [18] with slight modifications [14]. The hybridized probe was visualized with an ECL detection system (Amersham Biosciences). Fingerprinting images were analyzed with Fingerprinting™ II software (Bio-Rad Laboratories, Inc., Hercules, CA), and percent similarity among the isolates was determined according to the supplier’s instructions. To classify strains into the same family on the basis of their genotyping profiles, a similarity index of 70%, slightly more stringent than 65% used in a previous report [19] was chosen in this study. Normalization was performed using molecular weight standards and the IS*6110*-fingerprinting patterns of two isolates run on each gel. Isolates with fewer than five IS*6110* copies were excluded from the cluster analysis.

### DNA sequencing of INH resistance-related genes

The *furA*-*katG* operon and its upstream region were amplified by PCR using the specific primers and conditions described previously [16]. The primers used were -129furA (5′-GCTCATCGGAACATACGAAG-3′) and katG +50 (5′-GTGCTGCGGCGGGTTGTGGTTGATCGGCGG-3′). The *fabG1-inhA* operon and the upstream region of the *fabG1-inhA*operon were also amplified using previously reported primers [16]: -200fabG1 (5′-TTCGTAGGGCGTCAATACAC-3′) and inhA +40 (5′-CCGAACGACAGCAGCAGGAC-3′). PCR products were used as templates for direct DNA sequencing. To detect mutations, DNA sequences were compared with those of H37Rv using Genetyx-Mac, version 14.0.2 (Genetyx Corporation, Tokyo, Japan).

### Statistical analysis

Chi-square tests were used to compare proportions between two groups. Kappa statistics were used to determine the agreement between two tests. The following guidelines were used to interpret kappa coefficients: <0, poor agreement; 0–0.20, slight; 0.21–0.40, fair; 0.41–0.60, moderate; 0.61–0.80, good; and 0.81–1.00, very good. *P* values <0.05 were considered statistically significant, unless otherwise noted. The Bonferroni correction was used when comparing the results for multiple drugs. JMP version 9.0.0 (SAS Institute, Inc., Cary, NC, USA) statistics software was used for analysis.

## Results

### Clinical isolates from AFB-positive sputum

Clinical isolates were collected from 346 consecutive previously-untreated patients with AFB-positive active TB in Hanoi, Vietnam. Of these patients, 270 (78.0%) were male, and their median age was 38 years (range: 17–84 years). Coinfection with HIV was found in 31 patients (9.0%).

### Drug susceptibility profiles of MTB isolates

Sputum samples from 346 smear-positive patients were cultured, from which 339 MTB isolates (98.0%) were obtained. DST information for seven patients was not available. Among these, 127 (37.5%) were resistant to at least one of the four drugs tested; 93 (27.4%), 19 (5.6%), 96 (28.3%), and 11 (3.2%) isolates were resistant to INH, RMP, SM, and EMB, respectively; and 17 (5.0%) were multidrug-resistant (MDR) strains (Table 1). The PZase assay revealed that 8 (2.4%) of the 339 isolates were negative for PZase and were considered to be resistant to PZA (data not shown).

**Table 1:** Drug susceptibility profiles of MTB isolates from previously-untreated patients

**Patterns**		**n**	**% (95% CI)**
Sensitive with all drugs		212	62.5 (57.3 - 67.5)
Any resistance	Subtotal	127	37.5 (32.5 - 42.7)
	INH	93	27.4 (23.0 - 32.4)
	RMP	19	5.6 (3.6 - 8.6)
	SM	96	28.3 (23.8 - 33.3)
	EMB	11	3.2 (1.8 - 5.7)
MDR		17	5.0 (3.2 - 7.9)

N = 339 isolates.

INH concentration (0.2 μg/mL).

Eighty-eight INH-sensitive and 64 INH-resistant strains were randomly selected. LiPAs for RMP and INH were performed to confirm consistency with the results of culture-based DST and to identify profiles of genetic mutations associated with resistance to these drugs. Agreement between LiPAs and conventional DST was good or very good (kappa = 0.80 for RMP and kappa = 0.84 for INH 0.2 μg/mL; table not shown).

Mutations for RMP included *rpoB*:H526D, H526Y, S526D, and S531L. Only 11 isolates had one of these mutations, and 4 undefined RMP mutations were also observed in our study.

Mutations for INH were mostly *katG*:S315T (data not shown). This was more widely confirmed by subsequent DNA sequencing around the *katG* and *inh* genes (Additional file 1). LiPA for PZA was compared with the results of the PZase assay; their consistency was moderately high (kappa = 0.55; table not shown). LiPA for FQ identified only 1 mutated strain carrying *gyrA*:A90V.

### *IS6110*-probed RFLP and drug susceptibility

**Figure 1:**
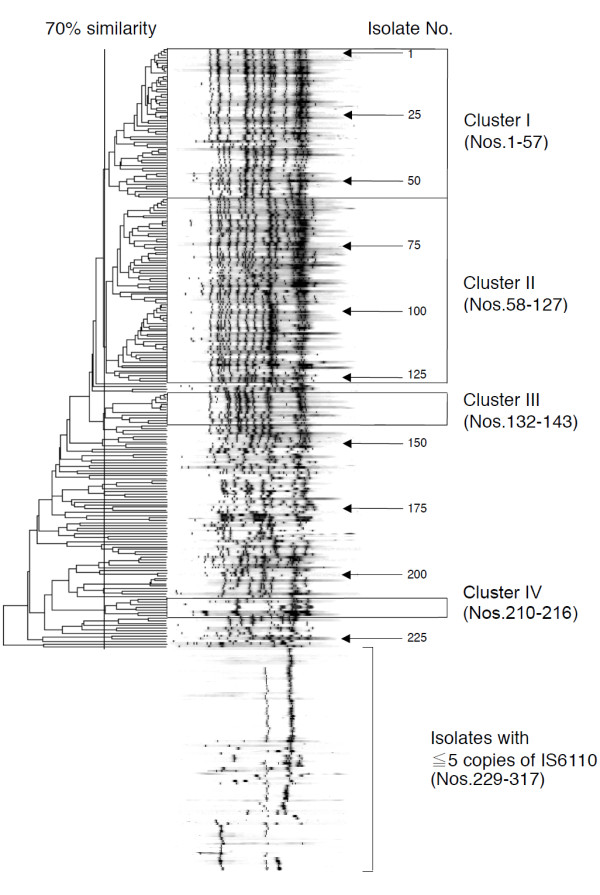
***IS6110*-probed fingerprinting patterns (N = 317).**

Sufficient DNA was extracted from 317 of the 339 isolates. Their IS*6110*-probed fingerprinting patterns are shown in Figure 1. Four clusters were identified when defined by >70% similarity in their RFLP patterns. These clusters accounted for 146 (46.1%) of the total isolates: 57 (18.0%) in cluster I; 70 (22.1%) in cluster II; 12 (3.8%) in cluster III; and 7 (2.2%) in cluster IV. Each cluster consisted of isolates collected from at least three districts in the city of Hanoi (data not shown).

Within these four clusters, 55 and 49 (37.7% and 33.6%) of the isolates were resistant to INH at 0.2 μg/mL and 1.0 μg/mL, respectively; these proportions were significantly higher than the 19.9% and 17.0% among the non-clustered isolates (*P* = 0.0004 and *P* = 0.0006, respectively; Table 2). In fact, in the small clusters III and IV, the majority of the isolates were highly resistant to INH at 1.0 μg/mL (Additional file 1: Table S1). Although the proportions of isolates in these clusters that were resistant to RMP and other drugs also tended to be higher than those of non-clustered isolates, these differences were not significant, based on multiple comparisons statistical testing (Table 2). In addition, there were no significant associations between the clusters and specific *rpoB* mutations (data not shown).

**Table 2:** The relationship between drug resistance and clustering of the clinical isolates in Hanoi

**Resistant to**	**MTB isolates**		P value*
	**Clustered**	**Non-clustered**	
	**N = 146**	**N = 171**	
INH (0.2 μg/mL)	55 (37.7%)	34 (19.9%)	0.0004
INH (1.0 μg/mL)	49 (33.6%)	29 (17.0%)	0.0006
RMP	13 (8.9%)	6 (3.5%)	0.0437
SM	49 (33.6%)	41 (24.0%)	0.0592
EMB	7 (4.8%)	4 (2.3%)	0.2338

*P values by chi-square test. P < 0.01 was regarded as statistically significant after considering multiple comparisons.

### Gene mutations responsible for INH resistance

Among all the INH-resistant isolates noted above, possible mutations within the *furA*-*katG* operon, the *fabG1*-*inhA* operon, and their upstream regions were investigated by PCR-based nucleotide sequencing (Additional file 1: Table S1).

Of 89 INH (0.2 μg/mL)-resistant isolates, 76 (85.4%) carried a g944c mutation (AGC to ACC) that caused an S315T amino acid substitution in the *katG* gene and 70 (92.1%) of the isolates that carried the g944c mutation were highly resistant to INH at 1.0 μg/mL. Furthermore, g204c mutations in the *furA* operon were detected in 13.6% of all the isolates and were frequently accompanied by the g944c mutation in the *katG* gene, although this variant itself was not directly associated with culture-based INH resistance (*P* = 0.072; table not shown). In the upstream region of the *fabG1*-*inhA* operon, c-15t was observed in 7 isolates, and minor variations with <1% were observed within the *fabG1*-*inhA* operon. The combination of a g944c mutation in the *katG* gene with c-15t in the *inhA* promoter was observed only in 1 isolate.

## Discussion

We investigated the drug susceptibility profiles of clinical isolates obtained from previously untreated patients with active pulmonary TB in Hanoi, the northern largest city in Vietnam, and found that a quarter of these isolates were highly resistant to INH, most of which had a single S315T mutation in the *katG* gene. These isolates with primary resistance to INH were enriched in the clusters identified by RFLP. They probably originated from a few genetically related clones and were recently transmitted into and spread within this area of Vietnam.

Among the isolates with resistance to the first-line drugs tested in this study, a high proportion of primary INH resistance (27.4%) was rather characteristic. This resistance level was higher than the average (19.1%) obtained during a 2006 nation-wide survey [5]. Such a high level of primary resistance is a serious concern because INH is a key drug by which newly diagnosed TB patients can be successfully treated. In Vietnam, culture-based DST has not yet been routinely performed for previously untreated patients, and the standard regimen for these patients remained 2S(E)HRZ/6HE for a long time [20]. In the years when RMP-based treatment was not easily accomplished during the maintenance phase in areas with inadequate resources, this regimen had certain significance and was thus endorsed by the WHO until recently [21].

However, when DST results are unknown and the above standard regimen is used for INH-resistant TB, treatment during the maintenance phase is no more than EMB monotherapy, which could increase the chances of failure, early relapse, and additional drug resistance [21,22]. Prescribing 2RHEZ/4RHE, a regimen that includes 6 months of RMP, has also been recently approved by the national TB program in Vietnam. Because treatment outcomes are largely affected by locale-specific factors, including patient adherence to the regimen and drug resistance profiles of the prevailing strains, further studies will be needed to confirm the optimal regimens in Vietnam [23].

To reduce the likelihood of failure, relapse, and additional acquired drug resistance in major cities, updating clinical laboratories for DST is an urgent need [21]. In addition to DST for first-line drugs, detecting resistance to second-line drugs, including FQ, has also become important recently [24], although the proportion appeared to be low (<1%) in our study. Even in a resource-poor setting, as per timely DST results, health care staff should treat and intensively follow up those patients with drug-resistant TB with the aim of complete cure in most of these cases and to prevent further spread of MDR-TB and generation of extensively drug-resistant TB.

Genetic analysis of our MTB isolates demonstrated that >85% of the INH resistance (92% with high-level resistance) was caused by a S315T mutation in the *katG* gene. The predominance of this mutation in INH resistance has been observed in most of the areas with high TB prevalence, although the proportion (85%) in our study was relatively high compared with what was reported in other studies [25-27]. Continuous use of INH may cause additional mutations and induce higher levels of resistance [27,28]. Rapid detection of INH resistance at an early phase is important to break this chain of acquiring additional resistance. Predominance of the S315T mutation is potentially advantageous for providing molecular DST in a resource-limited setting because it might entice manufacturers to develop a simplified, maintenance-free genetic test specialized for detecting the relevant mutations at a reasonably low cost [29].

RFLP analysis demonstrated that primary resistance to INH was more often observed in clustered isolates than in non-clustered isolates. Resistance to other drugs also appeared to be associated with these clustered isolates, although the tendencies were not as clear as that for INH. This indicates that expansion of INH-resistant isolates presumably originated from a few genetically related clones and that they were transmitted into the city of Hanoi and they spread widely within a relatively short period. Rapid expansion of genetically related strains may also explain why a single INH-resistant mutation, S315T, was predominantly detected in this area.

In our study, other genotype data for these clinical isolates were not available. However, in Vietnam, particularly in the southern region, two families of strains, designated the Beijing genotype and a presumably indigenous East-African Indian (EAI) genotype, are known to be predominant [30]. According to the literature, strains with ≥15 IS*6110* copies may indicate typical Beijing strains [31], whereas most of the EAI strains in Vietnam have <5 copies [30]. The copy numbers and RFLP pattern profiles in large cluster II in our study were definitely consistent with those of Beijing strains, whereas approximately one-fourth of the isolates had a few copies that are observed in EAI. IS*6110* copy numbers in other clusters were those between these two families. An earlier study demonstrated that copy numbers of northern strains in Vietnam were relatively smaller than those of southern strains in which typical Beijing genotypes are frequently observed [6]. We will need to further characterize the strains originating from the Hanoi area in future genotypic studies.

Our study had some limitations. The isolates analyzed in this study were collected from sputum smear-positive patients who visited TB clinics. Therefore, we may have only extracted features of MTB isolates from moderate to severe pulmonary TB cases. Nevertheless, understanding the current status of highly transmissible smear-positive TB is a priority for TB control because Vietnam is one of the high TB burden countries.

## Conclusions

High levels of primary resistance to INH and emerging RMP resistance may be closely related to the problems of a rapidly developing city, such as the distribution of young workers with low incomes, undernutrition, poor hygiene, and crowding in a densely populated urban area with a floating population. Private acquisition and inappropriate use of anti-TB drugs through unofficial distribution routes are also difficult to manage in a large city such as Hanoi. It will be necessary to curb the transmission of drug-resistant MTB by considering effective counter measures. We will need to carefully monitor these trends further and search for the origins and transmission routes of these Southeast Asian MTB strains.

## Competing interests

The authors declare that they have no competing interests.

## Authors’ contributions

NVH supervised on-site implementation of this study and drafted and revised the manuscript. HA performed the experiments and participated in technical transfer and supervision. TTBT carried out the drug susceptibility tests. TK performed the experiments. NTLH supervised on-site implementation of this study and drafted and revised the manuscript. SS and PHT monitored on-site data collection. LTL conceived and supervised this study. NK conceived the study, analyzed and interpreted data, and drafted and revised the manuscript. All authors read and approved this manuscript.

## Authors’ information

NVH is the head of the National Mycobacteria Reference Laboratory in the National Lung Hospital, Vietnam, and a senior expert on MTB. HA has extensive experience in MTB molecular analysis techniques. TTBT is the deputy head of the National Mycobacteria Reference Laboratory and has extensive experience in drug susceptibility testing. TK has experience in MTB molecular analysis techniques. NTLH has experience in clinical research. SS has experience in field studies. PHT is the director of the Hanoi Lung Hospital and is an expert on TB management. LTL is the vice director of the Hanoi Department of Health and is a senior expert on TB management. NK is the head of the Department of Pathophysiology and Host Defense, the Research Institute of Tuberculosis, Japan, and has experience in clinical research, data analysis, and TB control.

## Supplementary Material

Additional file 1: Table S1 Drug susceptibility testing and DNA sequencing of *M. tuberculosis* clinical isolates (N = 317). Click here for file
